# Quality of life in South East Asian patients who consult for dyspepsia: Validation of the short form Nepean Dyspepsia Index

**DOI:** 10.1186/1477-7525-7-45

**Published:** 2009-05-23

**Authors:** Sanjiv Mahadeva, Hwee-Lin Wee, Khean-Lee Goh, Julian Thumboo

**Affiliations:** 1 Division of Gastroenterology, Department of Medicine, Faculty of Medicine, University of Malaya, Kuala Lumpur, Malaysia; 2 Department of Rheumatology and Immunology, Singapore General Hospital, Singapore; 3 Department of Pharmacy, National University of Singapore, Singapore; 4 Department of Medicine, Yong Loo Lin School of Medicine, National University of Singapore, Singapore

## Abstract

**Background:**

Treatment objectives for dyspepsia include improvements in both symptoms and health-related quality of life (HRQoL). There is a lack of disease-specific instruments measuring HRQoL in South East Asian dyspeptics.

**Objectives:**

To validate English and locally translated version of the Short-Form Nepean Dyspepsia Index (SF-NDI) in Malaysian patients who consult for dyspepsia.

**Methods:**

The English version of the SF-NDI was culturally adapted locally and a Malay translation was developed using standard procedures. English and Malay versions of the SF-NDI were assessed against the SF-36 and the Leeds Dyspepsia Questionnaire (LDQ), examining internal consistency, test-retest reliability and construct validity.

**Results:**

Pilot testing of the translated Malay and original English versions of the SF-NDI in twenty subjects did not identify any cross-cultural adaptation problems. 143 patients (86 English-speaking and 57 Malay speaking) with dyspepsia were interviewed and the overall response rate was 100% with nil missing data. The median total SF-NDI score for both languages were 72.5 and 60.0 respectively. Test-retest reliability was good with intraclass correlation coefficients of 0.90 (English) and 0.83 (Malay), while internal consistency of SF-NDI subscales revealed α values ranging from 0.83 – 0.88 (English) and 0.83 – 0.90 (Malay). In both languages, SF-NDI sub-scales and total score demonstrated lower values in patients with more severe symptoms and in patients with functional vs organic dyspepsia (known groups validity), although these were less marked in the Malay language version. There was moderate to good correlation (*r *= 0.3 – 0.6) between all SF-NDI sub-scales and various domains of the SF-36 (convergent validity).

**Conclusion:**

This study demonstrates that both English and Malay versions of the SF-NDI are reliable and probably valid instruments for measuring HRQoL in Malaysian patients with dyspepsia.

## Introduction

Dyspepsia refers to a collection of recurrent upper gastrointestinal symptoms that is common world-wide [1]. Although usually not life-threatening, the impact of this condition in terms of frequency of medical consultation, drug utilisation and work absenteeism [2,3] has been shown to be considerable. As most patients with dyspepsia have functional disease, the treatment of which remains unsatisfactory at present [4], health related quality of life (HRQoL) measurement has become an important clinical objective in the assessment of new therapies for this condition [5].

Disease-specific HRQoL instruments, as opposed to generic HRQoL instruments, are better able to detect HRQoL changes for specific diseases and hence are more clinically useful for detecting the effectiveness of various treatments in these conditions [6]. Although several HRQoL instruments for dyspepsia currently exist [7-9], their applicability has been limited by insufficient specificity for dyspepsia alone or lack of brevity [10]. The Short-Form Nepean Dyspepsia Index (SF-NDI) is a brief, multi-dimensional dyspepsia-specific HRQoL measure developed in the English language. It has been shown to be a responsive and sensitive instrument for the measurement of dyspepsia-related HRQoL in several different English-speaking populations around the world [10].

The prevalence of dyspepsia in Malaysia, a country with an ethnically and culturally diverse population typical of the South East Asian region, is estimated between 27 – 35% [11,12]. Recent community-based studies have also demonstrated high medical consultation rates for Malaysian patients with dyspepsia [12], although many are known to have functional disease as well [13]. To our knowledge, no existing HRQoL instrument has been validated nor new tools developed to measure HRQoL in Malaysian patients with dyspepsia. The English language and Malay, the official and national language, are the 2 commonest languages spoken in this country. In this study, we thus aimed to translate and cross-culturally adapt both English and Malay versions of the SF-NDI, and to validate both language versions in Malaysian dyspeptics as a prelude to future clinical trials and evaluations of medical therapy for dyspepsia in this population.

## Methods

### Subjects

Consecutive adult outpatients with dyspepsia attending the Gastroenterology clinic of the University Malaya Medical Centre, a tertiary teaching institution, were invited to participate in the study. Functional or non-ulcer dyspepsia was defined as dyspepsia with normal or minor endoscopic features, whilst dyspeptics who had endoscopic findings of duodenal erosions, peptic ulcer disease or erosive oesophagitis were defined as organic dyspepsia. Patients were interviewed by a trained research assistant using identical English or Malay instruments and assessing the period of medical consultation and socio-economic-demographic status. Local institutional ethics committee approval was obtained to conduct this study.

### Instruments

*The Short Form (SF) Nepean Dyspepsia Index *is a 10-item questionnaire with 5 sub-scales each examining the influence of dyspepsia on domains of health in patients, namely tension/anxiety, interference with daily activities, disruption to regular eating/drinking, knowledge towards/control over disease symptoms and interference with work/study, with each sub-scale containing two items [10]. Each item is measured by a 5-point Likert scale ranging from 0 (not at all or not applicable), 1 (a little), 2 (moderately), 3 (quite a lot) to 4 (extremely). Individual items in each sub-scale are aggregated to obtain a score range from 0 (lowest HRQoL score) to 100 (highest HRQoL score) as per the developers' original calculation formula [14]. A total, overall SF-NDI total score is obtained using the mean of 5 subscale scores.

*The Short Form 36 (SF-36) *is an established generic health-related HRQoL instrument which comprises 36 questions in eight different subscales: physical functioning, physical role limitations, bodily pain, general health perceptions, vitality, social functioning, emotional role limitations, mental health and 2 composite scores – Physical Component (PCS) and Mental Component Scores (MCS) [15]. The maximum score of 100 indicates the best possible health state. This instrument has previously been translated and validated in the Malaysian population and shown to be a reliable measure of general HRQoL status [16].

The *Leeds Dyspepsia Questionnaire *(LDQ), is an eight item symptom-based questionnaire assessing the severity of dyspepsia through the frequency and severity of various upper G.I. symptoms, namely upper abdominal pain/discomfort, heartburn, regurgitation, dysphagia, belching, nausea, vomiting and post-prandial distension/early satiety [17]. The total score ranges from 0 – 40, with lower values indicating less and higher values more severe dyspepsia. A score of 15 or more has been defined by the developers as indicative of severe dyspepsia. The questionnaire has previously been validated in the Malaysian population and shown to be reliable in assessing dyspepsia amongst Malaysians [18].

### Cultural validation of the English version of the SF-NDI

Cross-cultural adaptation of the English version of the SF-NDI was performed in 10 English-speaking healthy subjects of varied age and educational backgrounds. In-depth cognitive interviews were conducted to determine appropriateness of the original English version in Malaysian adults. Alterations were made to the original instrument if particular words or sentences were not understood and further cognitive debriefing performed until a conceptually and semantically acceptable English version of the SF-NDI was developed for this population.

### Translation of the Short Form Nepean Dyspepsia Index (SF-NDI)

A Malay version of the SF-NDI was developed using standard forward-back translation. Two independent forward translations (source English version to target Malay version) were first produced with the aim of achieving equivalence in concepts (i.e. conceptual equivalence) and meaning (i.e. semantic equivalence), from which a consensus forward Malay translation was obtained, with differences resolved through discussion. Any problems in the forward translation were documented and two independent back translations (Malay to English) were then produced from the consensus forward translation as a quality check. Following approval by the original instrument developer, a consensus Malay version was derived and cognitive interviews conducted with ten subjects of varied age and educational backgrounds. If necessitated by results of cognitive debriefing, it was planned to perform an iterative process of review by translators followed by further cognitive debriefing till a conceptually and semantically acceptable Malay translation of the SF-NDI was developed.

### Validation of SF-NDI

Psychometric properties of both English and Malay versions of the SF-NDI were evaluated by assessing their internal consistency, reliability, validity, sensitivity and frequency of missing data. Internal consistency was assessed using Cronbach's alpha, with a value of 0.7 being taken as adequate for group comparisons. Test-retest reliability of SF-NDI was evaluated by administering the SF-NDI twice to the same subjects, 2-weeks apart, and assessing the consistency of scores obtained on these two occasions. The second interview was conducted over the phone by a trained research assistant. Validity of the SF-NDI was determined by assessing whether the sub-scales and utility score actually measured the desired attribute (i.e. construct validity). This process included convergent, and known-groups construct validity. For convergent validity, sub-scales of the SF-NDI were correlated with similar dimensions of an established instrument, the SF-36. Known-groups validity involved testing 12 a-priori hypothesis that all five SF-NDI sub-scales and the summary total score would demonstrate lower values in patients with more severe dyspeptic symptoms [9,10,19] and in those with functional compared to organic disease [20-22].

### Statistics

Hypothesized trends were tested using Chi-square or Mann-Whitney tests, or Spearmans' correlation coefficient where appropriate. Strong, moderate and weak correlations were defined as > 0.60, 0.30 – 0.60 and < 0.30 respectively [23]. Test-retest reliability was assessed using intraclass correlation coefficients (ICC), with a desired value of > 0.7 [24]. Statistical significance for hypothesis fulfillment was defined as a p value of < 0.05. Data were analysed with SPSS for windows (version 12, SPSS Inc, IL, USA).

## Results

### Cross-cultural adaptation of the English SF-NDI

Cognitive debriefing of the original English version of the SF-NDI was conducted on 10 English-speaking healthy subjects – five were aged below 50 years and six had had tertiary education. No difficulties were encountered by all ten subjects with understanding phrasing of the original English SF-NDI items and no changes were made prior to validation.

### Adaptation of the Malay SF-NDI

A Malay translation of the SF-NDI was produced according to the standard protocol detailed above. Cognitive debriefing of this translated SF-NDI was conducted on 10 subjects – 7 female nurses, 2 female clerks and 1 male clerk, all of Malay ethnicity and with 10 or less years of education. No difficulties were encountered by all ten subjects with regards to understanding phrasing of the Malay SF-NDI items and therefore no further changes were made prior to use in the validation study.

### Patient characteristics

A total of 143 patients with dyspepsia were interviewed between October 2007 to December 2008, with a 100% response rate. 86 patients were interviewed in English and 57 were interviewed in Malay. Their socio-demographic characteristics are summarized in Table 1. The ages of patients were varied in both language categories, with a mean of 56.2 ± 14 and 43.3 ± 14.9 years amongst English and Malay speaking patients respectively. The male:female ratio was similar (Table 1) and ethnicity varied in both language categories as follows: English-speaking – 6 (7.0%) Malays, 40 (46.5%) Chinese, 38 (44.2%) Indians and Malay-speaking 22 (38.6%) Malays, 3 (5.3%) Chinese, 27 (47.4%) Indians. Education levels in both language categories were similar with 89.5% of patients having 12 or more years of education, but more patients were retirees in the English-speaking group (44.2% vs 28.1%). The majority of cases (68.6% English-speaking, 77.2% Malay-speaking) had a diagnosis of functional dyspepsia (Table 1). The median period of medical (either primary care or hospital specialist) consultation (3 months in both language categories) and the median LDQ score in patients (9 in English-speaking, 13 in Malay-speaking), indicated persistent recurrent symptoms in the study group.

**Table 1:** Characteristics and demography of Malaysian patients with dyspepsia in the study

	English speaking n = 86	Malay speaking n = 57
Mean age (SD)	56.2 (14)	43.3 (14.9)

Gender (Male: Female)	1:1.10	1:1.04

Ethnicity:		
Malay	6 (7.0%)	22 (38.6%)
Chinese	40 (46.5%)	3 (5.3%)
Indian	38 (44.2%)	27 (47.4%)
Native	2 (2.3%)	5 (8.8%)

Education level:		
Primary	9 (10.5%)	6 (10.5%)
Secondary	51 (59.3%)	32 (56.1%)
Tertiary	26 (30.2%)	19 (33.3%)

Marital status:		
Unmarried/separate/divorced	17 (19.8%)	13 (22.8%)
Married	61 (70.9%)	43 (75.4%)
Widowed	8 (9.3%)	1 (1.8%)

Employment status:		
Employed	31 (31.0%)	28 (49.1%)
Unemployed/homemaker	12 (13.9%)	8 (0.14%)
Retired	38 (44.2%)	16 (28.1%)

Diagnosis:		
Functional dyspepsia	59 (68.6%)	44 (77.2%)
Peptic ulcer disease	5 (5.8%)	7 (12.3%)
Gastroesophageal reflux disease	22 (25.6%)	6 (10.5%)

Length of dyspeptic symptoms (months) (median; interquartile range)	6.5 (4 – 20)	12 (3.3 – 24)

Period of medical consultation (months) (median; interquartile range)	3 (5.5 – 18.3)	3 (10.0 – 20.0)

Leeds Dyspepsia Questionnaire score (median; interquartile range)	9 (15 – 20)	13 (17 – 24)

### Domain (sub-scale) and summary (total) values of English and Malay versions of the SF-NDI

**Figure 1:**
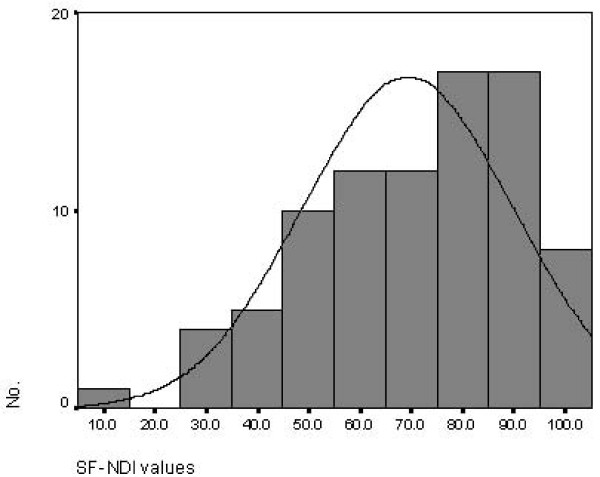
**Histogram of SF-NDI total score values among English-speaking patients (n = 86)**.

There were no missing data for all variables. In the English-speaking group, the median total SF-NDI score was 72.5, with ceiling and floor effects of 5.0 – 100.0. A histogram (Figure 1) revealed a skewed distribution (skewness -.635) of scores with an interquartile range (IQR) from 55.0 to 85.0. Median scores for each SF-NDI sub-scale were as follows: tension/anxiety 75.0 (IQR 46.9 – 75.0), interference with daily activity 75.0 (IQR 50.0 – 100.0), eating/drinking 75.0 (IQR 37.5 – 87.5), knowledge/control 75.0 (IQR 62.5 – 87.5), and work/study 75.0 (IQR 62.5 – 100.0).

**Figure 2:**
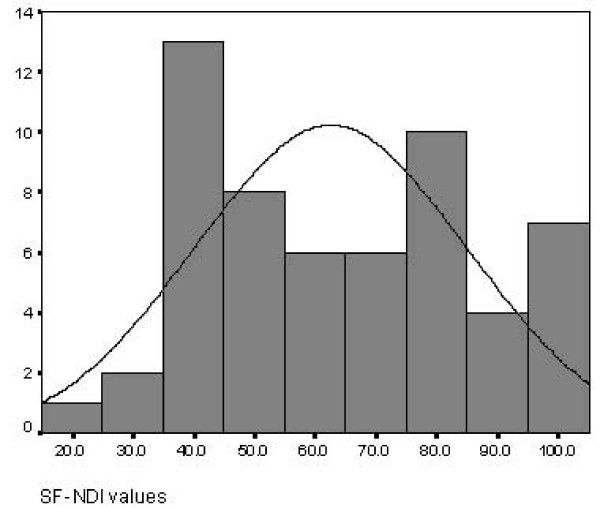
**Histogram of SF-NDI total score values among Malay-speaking patients (n = 57)**.

Among patients who were interviewed in Malay, the median SF-NDI score was 60.0, with ceiling and floor effects of 22.5 to 100.0. A histogram chart (Figure 2) revealed a near normal distribution (skewness 0.078). Median scores for each SF-NDI sub-scale were as follows: tension/anxiety 62.5 (IQR 37.5 – 75.0), interference with daily activity 75.0 (IQR 43.8 – 87.5), eating/drinking 50.0 (IQR 37.5 – 81.3), knowledge/control 75.0 (IQR 50.0 – 87.5), and work/study 62.5 (IQR 43.7 – 75.0).

### Reliability of both English and Malay versions of the SF-NDI

Cronbach's α was used to assess internal consistency for both language versions of the SF-NDI. In the English version, α values for each sub-scale were as follows: tension/anxiety 0.84, interference with daily activity 0.83, eating/drinking 0.85, knowledge/control 0.88 and work/study 0.85. In the Malay version, α values for each sub-scale were as follows: tension/anxiety 0.84, interference with daily activity 0.87, eating/drinking 0.84, knowledge/control 0.90 and work/study 0.83.

120 (73 English-speaking and 47 Malay-speaking) patients participated in the follow-up telephone interview, which was conducted at a median of 16 days (range 13 – 18) after the original interview. In the English speaking group, ICC between baseline and follow up SF-NDI total (summary) scores was high at 0.90 (95% CI = 0.85 – 0.94), demonstrating excellent test-retest reliability, while English NDI sub-scale ICC values were as follows: tension/anxiety 0.91, interference with daily activity 0.88, eating/drinking 0.88, knowledge/control 0.86 and work/study 0.95. In the Malay speaking group, ICC between baseline and follow up SF-NDI total (summary) scores was 0.83 (95% CI = 0.69 – 0.90), equally demonstrating adequate test-retest reliability, while Malay NDI sub-scale ICC values were as follows: tension/anxiety 0.72, interference with daily activity 0.77, eating/drinking 0.78, knowledge/control 0.83 and work/study 0.91.

### Validity of both English and Malay versions of the SF-NDI

Known-groups validation was assessed in both language instruments separately. In the English version, 8/12 hypotheses relating to SF-NDI sub-scales were fulfilled (Table 2). All five sub-scales had significantly lower HRQoL scores in patients with severe dyspeptic symptoms compared to those with mild symptoms, as determined by the LDQ score. Lower scores were noted for the SF-NDI "tension", "interference", "work" sub-scales in patients with functional dyspepsia compared to organic cases and for the overall summary score (Table 2). In the Malay version, 4/12 hypotheses were fulfilled with another four demonstrating trends in the hypothesized direction (Table 3).

**Table 2:** Known groups construct validity of the English version of the SF-NDI sub-scales (n = 86)

	Dyspepsia severity*			Dyspepsia aetiology		
			
SF-NDI sub-scale scores	Mild n = 23	Severe n = 63	p #	Organic n = 28	Functional n = 58	p #
Tension (median; range)	87.5 (50.0–100.0)	62.5 (0–100.0)	< 0.001	75.0 (25.0–100.0)	62.5 (0–100.0)	0.05

Interference (median; range)	100.0 (50.0–100.0)	75.0 (0–100.0)	< 0.001	87.5 (50.0–100.0)	75.0 (0–100.0)	0.01

Eating/drinking (median; range)	87.5 (37.5–100.0)	62.5 (0–100.0)	< 0.001	75.0 (37.5–100.0)	75.0 (0–100.0)	0.05

knowledge/control (median; range)	87.5 (75.0–100.0)	62.5 (0–100.0)	< 0.001	75.0 (0–100.0)	75.0 (12.5–100.0)	0.12

work/study (median; range)	100.0 (62.5–100.0)	75.0 (0–100.0)	< 0.001	87.5 (25.0–100.0)	75.0 (0–100.0)	0.02

Total (median; range)	90.0 (62.5–100.0)	65.0 (5.0–97.5)	< 0.001	78.8 (50.0–100.0)	68.8 (5.0–100.0)	0.02

* Mild = LDQ score < 15; Severe = LDQ score ≥ 15

# Mann-Whitney U test

**Table 3:** Known groups construct validity of the Malay version of the SF-NDI sub-scales (n = 57)

	Dyspepsia severity*			Dyspepsia aetiology		
			
SF-NDI sub-scale scores	Mild n = 11	Severe n = 46	p #	Organic n = 13	Functional n = 44	p #
Tension (median; range)	75.0 (25.0–100.0)	56.3 (0–100.0)	0.15	50.0 (0–100.0)	62.5 (12.5–100.0)	0.53

Interference (median; range)	75.0 (37.5–100.0)	62.5 (0–100.0)	0.14	75.0 (25.0–100.0)	75.0 (0–100.0)	0.76

Eating/drinking (median; range)	75.0 (37.5–100.0)	50.0 (0–100.0)	0.04	50.0 (25.0–100.0)	56.3 (0–100.0)	0.62

knowledge/control (median; range)	87.5 (37.5–100.0)	68.8 (25–100.0)	0.03	75.0 (25–100.0)	68.8 (25–100.0)	0.47

work/study (median; range)	75.0 (12.5–100.0)	62.5 (0–100.0)	0.02	62.5 (25.0–100.0)	62.5 (0–100.0)	0.85

Total (median; range)	77.5 (35.0–100.0)	56.3 (22.5–100.0)	0.05	62.5 (27.5–100.0)	58.8 (5.0–100.0)	0.91

* Mild = LDQ score < 15; Severe = LDQ score ≥ 15

# Mann-Whitney U test

Convergent validity demonstrated moderate to good correlation between English and Malay versions of the SF-NDI sub-scales with various domains of the SF-36 (Additional file 1). In the English version, relevant sub-scales and the total summary score of the SF-NDI showed moderate correlations with various SF-36 domains ranging from general health (*r *= 0.37, p < 0.001) and bodily pain (*r *= 0.45, p < 0.001) to social functioning (*r *= 0.51, p < 0.001) and mental component summary score (*r *= 0.61, p < 0.001). The Malay version of the SF-NDI total summary score demonstrated similar moderate correlations with SF-36 domains such as role physical (*r *= 0.32, p < 0.001), bodily pain (*r *= 0.54, p < 0.001), social functioning (*r *= 0.33, p < 0.05) and vitality (*r *= 0.30, p < 0.001) (Additional file 1).

## Discussion

It is well recognized that the outcome of dyspepsia management is dependent on patients' perception of their well-being in relevant physical, emotional and social domains [4]. As such, the measurement of change in HRQoL in patients with dyspepsia has become an important treatment objective in addition to symptom improvement [5]. There is a lack of validated disease-specific instruments measuring HRQoL in South East Asians, a population with a high prevalence of dyspepsia and frequent medical consultation rates [12]. We have developed a Malay translation of the SF-NDI which is conceptually equivalent to the source version, and determined that the original English version is culturally suitable for English-speaking adults in this country. In this study, both the original English and Malay versions of the SF-NDI have been found to be acceptable and easily understood by Malaysian dyspeptics, and demonstrated to have good psychometric properties, suggesting that the SF-NDI is suitable for use in these patients.

The patient sample in this study was fairly representative of most dyspeptics seeking attention at secondary/tertiary care institutions. Most of the patients (72.9%) had functional dyspepsia, had had prolonged periods of medical consultation at both primary and secondary/tertiary care and moderately high LDQ scores, indicating persistence of symptoms. Twenty eight patients with predominant upper abdominal discomfort were diagnosed with reflux oesophagitis, and 12 patients with peptic ulcer disease were under follow up following a recent diagnosis would usually be discharged once ulcer healing and symptom improvement had been achieved.

In both English and Malay versions of the SF-NDI all five sub-scales of the SF-NDI were found to have high internal consistency and repeated measurements over a short period (i.e. test-retest reliability) showed high correlation, indicating the reliability of the instrument in this population. Patients with more severe dyspeptic symptoms (measured by the LDQ in this instance) have been known to demonstrate lower HRQoL scores [9,10,19]. Similarly, all SF-NDI sub-scales and total scores (both English and Malay versions) were lower in Malaysian patients with higher LDQ scores in this study. Although these differences did not reach statistical significance on a few of the subscales among the Malay-speaking patients, the trend was nevertheless consistent, that is lower SF-NDI sub-scale scores were associated with higher LDQ scores. This could suggest that the effect sizes on these scales are larger and requires a larger sample size to detect a statistically significant difference. Hence, the results are still suggestive of construct validity but need to be confirmed in future larger studies.

The increased association of life events and psychological disorders with functional dyspepsia compared to peptic ulcer disease [20,21], is presumed to be responsible for poorer HRQoL in patients who consult medical practitioners for their symptoms [22]. In this study, the SF-NDI total score were shown to be lower in Malaysian patients with functional dyspepsia compared to those with organic disease, supporting the construct validity of the instruments. Once again, the magnitude of this reduction in SF-NDI scores was less marked in the Malay version of the SF-NDI and the smaller sample size in this group might explain the lack of statistical significance.

Convergent validity of the English and Malay versions of the SF-NDI was further supported by moderate – good correlation with various domains of the SF-36, ranging from "general health" and "bodily pain" to "social functioning" and "vitality". These findings indicate that the SF-NDI, although relatively limited by 5 sub-scales, was sufficiently broad to examine various aspects of HRQoL, particularly in the sub-group of patients studied. Similar observations of the SF-NDI with generic HRQoL instruments such as the SF-36 [10] and SF-12 [25] have been noted in other validation studies.

Apart from the original developers validation of the instrument in European and North American adults with dyspepsia [10], only two other independent validation studies of the SF-NDI have been published [25,26]. In 104 Arabic patients with non-ulcer dyspepsia and gastro-esophageal reflux disease, an Arabic translation of the NDI was shown to have a high internal consistency (0.88 – 0.93) and adequate face and content validity. Convergence validity demonstrated moderate correlation with various domains of the SF-12 (a generic HRQoL instrument), similar to the findings from this study [25]. In another study of 52 Norwegian patients with food hypersensitivity disorder, a Norwegian translation of the SF-NDI was shown to be reliable and responsive to change [26]. It further demonstrated good correlation with two gastrointestinal symptom severity scales (the Gastrointestinal Symptom Rating Scale and the Ulcer Esophagitis Subjective Symptom Scale), lending the authors to suggest that the SF-NDI could be applied for a variety of gastrointestinal diseases and not dyspepsia alone. However, correlation analysis with other HRQoL instruments was not performed in this study, which limits the findings of this study somewhat.

Several other disease-specific HRQoL instruments have been developed for dyspepsia such as the quality of life in reflux and dyspepsia (QOLRAD) questionnaire [7], the Glasgow Dyspepsia Severity Score [8] and the Korean functional dyspepsia related quality of life (FD-QOL) scale [9]. The former 2 instruments have been quoted widely and are often used in dyspepsia-related trials in Western populations. The QOLRAD contains 25 items measuring various parameters relating to emotional, physical and social well-being, but a clear distinction between dyspepsia and reflux is not made and responsiveness to dyspepsia in particular has not been proven. The Glasgow Dyspepsia Severity score is an investigator-based global measure of dyspepsia and is not a comprehensive dyspepsia-specific HRQoL instrument. The Korean FD-QOL has been demonstrated to be appropriate for Korean patients with dyspepsia, but consists of 21 items and may be less easily translated to other languages. The brevity and simplicity of the SF-NDI in its' native English-form, on the other hand, lends well for translation into our local language and subsequent comprehension by adults in our population, which was demonstrated in our pilot study.

## Conclusion

Cultural and linguistic variations in different populations, an important variable influencing HRQoL [27], necessitate local cultural adaptation, translation and validation of established HRQoL instruments. In a representative South East Asian population with a significant prevalence of dyspepsia, we have demonstrated the validity and reliability of the SF-NDI in its original English form for measuring HRQoL in patients who consult for dyspepsia. Although found to be reliable in this study, the Malay version may require further evaluation in a larger study to confirm its validity. Further studies to examine other properties of the SF-NDI, such as its' responsiveness, i.e. the ability to detect change in HRQoL status, will be required in the future to demonstrate its' role in improving clinical care in our population.

## Abbreviations

HR-QOL: Health-related quality of life; SF-NDI: Short Form Nepean Dyspepsia Index; SF 36: Short Form 36; LDQ: Leeds Dyspepsia Questionnaire.

## Competing interests

The authors declare that they have no competing interests.

## Authors' contributions

SM and HLW designed the study, analysed and interpreted the data, and drafted the manuscript. KLG provided administrative support and contributed to data collection with SM. JT provided technical support and critical revision of the manuscript. All authors reviewed and approved final version of the manuscript.

## Supplementary Material

Additional file 1 **Spearman's correlation of SF-NDI sub-scales with SF-36 domains (convergent validity)**. The data provided represents correlation analysis between HRQOL domains of the SF-36 and both English and Malay versions of the SF-NDI. Significant correlations in particular domains have been highlighted. Click here for file
