# Supplementary material for: Quality of life in South East Asian patients who consult for dyspepsia: Validation of the short form Nepean Dyspepsia Index
Source: Health Qual Life Outcomes. 2009 May 23;7:45. doi: 10.1186/1477-7525-7-45 (PMC2693522; doi:10.1186/1477-7525-7-45)
Supplement: Additional file 1 — Spearman's correlation of SF-NDI sub-scales with SF-36 domains (convergent validity). The data provided represents correlation analysis between HRQOL domains of the SF-36 and both English and Malay versions of the SF-NDI. Significant correlations in particular domains have been highlighted. [file 1477-7525-7-45-S1.doc]

**Additional file 1** Spearman’s correlation of SF-NDI sub-scales with SF-36 domains (convergent validity)

|  | English version of the SF-NDI sub-scales | | | | | | Malay version of the SF-NDI sub-scales | | | | | |
| --- | --- | --- | --- | --- | --- | --- | --- | --- | --- | --- | --- | --- |
|  | Tension | Interference | Eating/  drinking | knowledge/control | Work/  study | Total | Tension | Interference | Eating/  drinking | knowledge/control | Work/  study | Total |
| PF | 0.01 | 0.25* | 0.15 | 0.18 | 0.24* | 0.23* | 0.21 | 0.30* | 0.11 | 0.29* | 0.27* | 0.28* |
| RP | 0.34** | 0.33** | 0.23* | 0.38** | 0.49* | 0.42** | 0.30** | 0.24 | 0.29* | 0.07 | 0.39* | 0.32** |
| BP | 0.38** | 0.37** | 0.29** | 0.44** | 0.36** | 0.45** | 0.49** | 0.43** | 0.55** | 0.33* | 0.48** | 0.54** |
| GH | 0.37** | 0.28* | 0.19 | 0.45** | 0.29** | 0.37** | 0.50** | 0.48** | 0.37** | 0.53** | 0.56** | 0.28* |
| VT | 0.40** | 0.35** | 0.31* | 0.35** | 0.48** | 0.46** | 0.21 | 0.28* | 0.21 | 0.15 | 0.34** | 0.30** |
| SF | 0.41** | 0.52** | 0.38** | 0.28** | 0.53* | 0.51** | 0.26 | 0.23 | 0.24 | 0.33* | 0.30* | 0.33* |
| RE | 0.38** | 0.34** | 0.31** | 0.41** | 0.42** | 0.46** | 0.30* | 0.27* | 0.18 | 0.08 | 0.33* | 0.27* |
| MH | 0.43** | 0.47** | 0.33** | 0.43** | 0.61** | 0.53** | 0.20 | 0.21 | 0.10 | 0.13 | 0.33* | 0.25 |
| PCS | 0.23* | 0.24* | 0.28* | 0.18* | 0.34* | 0.31* | 0.42** | 0.41** | 0.39** | 0.40** | 0.43** | 0.49** |
| MCS | 0.49** | 0.49** | 0.49** | 0.39** | 0.41** | 0.61** | 0.23 | 0.27* | 0.16 | 0.13 | 0.34* | 0.28* |

PF - physical functioning; RP – role limitation due to physical problems; BP - bodily pain; GH – general health; VT – vitality; SF – social functioning; RE – role limitation due to emotional problems; MH – mental health; PCS – physical component summary score; MCS – mental component summary score

* p < 0.05; ** p < 0.001
